# Parameter estimation for robust HMM analysis of ChIP-chip data

**DOI:** 10.1186/1471-2105-9-343

**Published:** 2008-08-18

**Authors:** Peter Humburg, David Bulger, Glenn Stone

**Affiliations:** 1 Department of Statistics, Macquarie University, North Ryde, NSW 2109, Australia; 2 CSIRO Mathematical and Information Sciences, North Ryde, NSW 2113, Australia

## Abstract

**Background:**

Tiling arrays are an important tool for the study of transcriptional activity, protein-DNA interactions and chromatin structure on a genome-wide scale at high resolution. Although hidden Markov models have been used successfully to analyse tiling array data, parameter estimation for these models is typically *ad hoc*. Especially in the context of ChIP-chip experiments, no standard procedures exist to obtain parameter estimates from the data. Common methods for the calculation of maximum likelihood estimates such as the Baum-Welch algorithm or Viterbi training are rarely applied in the context of tiling array analysis.

**Results:**

Here we develop a hidden Markov model for the analysis of chromatin structure ChIP-chip tiling array data, using *t *emission distributions to increase robustness towards outliers. Maximum likelihood estimates are used for all model parameters. Two different approaches to parameter estimation are investigated and combined into an efficient procedure.

**Conclusion:**

We illustrate an efficient parameter estimation procedure that can be used for HMM based methods in general and leads to a clear increase in performance when compared to the use of *ad hoc *estimates. The resulting hidden Markov model outperforms established methods like TileMap in the context of histone modification studies.

## 1 Background

High density oligonucleotide tiling arrays allow the investigation of transcriptional activity, protein-DNA interactions and chromatin structure across a whole genome. Tiling arrays have been used in a wide range of studies, including investigation of transcription factor activity [1] and of histone modifications in animals [2] and plants [3], as well as DNA methylation [4]. Analyses of these data are usually based either on a sliding window [1,5], or on hidden Markov models (HMMs) [6-8]. Other approaches have been suggested, e.g., by Huber *et al*. [9] and Reiss *et al*. [10], but are less common.

Parameter estimates for sliding window approaches as well as hidden Markov models are typically *ad hoc*. Although there are some notable exceptions in gene expression studies [8,11], no established procedures exist to obtain good parameter estimates from tiling array data, especially in the context of chromatin immunoprecipitation (ChIP-chip) experiments. Attempts have been made to obtain parameter estimates by integrating genome annotations into the analysis [12]. While this may provide good results when investigating transcriptional activity in well studied organisms, it is limited by the quality of available annotations. For ChIP-chip studies the required annotation data is unavailable. A method for the localisation of transcription factors from ChIP-chip experiments by Keleş [13] does obtain the required parameter estimates from the data and allows for variations in length of enriched regions.

Methods designed for the analysis of ChIP-chip data focus almost exclusively on the study of transcription factors [[6,7,10], and [13]]. While this is an important class of experiments, ChIP-chip studies are not limited to transcription factors, and the analysis of other ChIP-chip experiments may require new methods. One other area of active research that utilises ChIP-chip experiments is the study of histone modifications and chromatin structure [3]. Although both types of experiment employ the same technology, there are several important differences between them. Most importantly, the 147 bp of DNA bound by a histone complex are considerably longer than the typical transcription factor binding site, and the histone modifications of interest are expected to affect several neighbouring histones. Consequently the ChIP fragments derived from a transcription factor binding site all originate from a small region containing the given binding site while regions affected by histone modifications can be much longer than the ChIP fragments used. As a result of this, the data from histone modification experiments usually contain long regions of interest encompassing several non-overlapping ChIP fragments, rather than the short and relatively isolated peaks produced by transcription factor studies.

Here we consider the analysis of data from a histone modification study in *Arabidopsis *[3]. These data consist of four ChIP samples for histone H3 with lysine 27 trimethylation (H3K27me3) and four histone H3 ChIP samples that act as a control. The aim of this analysis is to identify and characterise regions throughout the genome that exhibit enrichment for H3K27me3. It is desirable to use a method which is specifically designed for the analysis of histone modifications or flexible enough to accomodate the varying length of enriched regions. Furthermore, the method should obtain all parameter estimates from the data without the use of genome annotations and be robust towards outliers. Amongst the methods discussed above TileMap [7] comes closest to these requirements. Although it was developed with transcription factor analysis in mind it is general enough that it should provide useful results for other ChIP-chip experiments. This is emphasised by its application to histone modification [3] and DNA methylation [4] data as well as transtription factor analysis [14,15]. TileMap obtains some, but not all, of the required parameter estimates from the data. To provide a method which meets the requirements oulined above we develop a two state HMM with *t *emission distributions. All parameter estimates for the model are obtained by maximum likelihood estimation using the Baum-Welch algorithm [16] and Viterbi training [17]. These methods have the advantage that no prior knowledge about parameter values is required and one need not rely on frequently unavailable genome annotations. To assess the performance of our model, we apply it to simulated and real data. Results are compared to those produced by TileMap. The remainder of this article is structured as follows. In Section 2 the hidden Markov model is developed and MLEs for all parameters are derived in Section 4. The performance of the resulting model is assessed in terms of sensitivity and specificity on simulated data in Sections 2.3.3–2.3.6. In Section 2.3.7 the model is used to analyse a public ChIP-chip data set [3] and results are compared to the original analysis of these data.

## 2 Results and discussion

Tiling array data consists of a series of measurements taken along the genome. Typically, microarray probes are designed to interrogate the genome at regular intervals. Design constraints such as probe affinity and uniqueness cause differences in probe density along the genome and can lead to large gaps between probes. Here we assume that the probe density is homogeneous except for a number of large gaps where the distance between adjacent probes is larger than max_gap. In the following analyses we use max_gap = 200 bp. This is identical to the value used by Zhang *et al*. [3], allowing for a direct comparison of results. Consider a ChIP-chip tiling array experiment with two conditions, a ChIP sample **X**_1 _targeting the protein of interest and a control sample **X**_2_. Each sample **X**_*l *_has *m*_*l *_replicates (*l *= 1, 2) providing measurements for *K *genomic locations. The measurements for each probe are summarised by the "shrinkage *t*" statistic [18]:

(1) $$y_{k}=\frac{{\bar{x}}_{1k}-{\bar{x}}_{2k}}{\sqrt{\frac{v_{1k}^{*}}{m_{1}}+\frac{v_{2k}^{*}}{m_{2}}}},$$

where $v_{lk}^{*}$ is a James-Stein shrinkage estimate of the probe variance obtained by calculating

(2) $$v_{lk}^{*}={\hat{\lambda }}_{l}^{*}s_{l\text{median}}^{2}+(1-{\hat{\lambda }}_{l}^{*})s_{lk}^{2},$$

and $s_{lk}^{2}$ are the usual unbiased empirical variances and ${\hat{\lambda }}_{l}^{*}$ is the estimated optimal pooling parameter

(3) $${\hat{\lambda }}_{l}^{*}=\min\left(1,\frac{\sum_{k=1}^{K}\overset{︿}{\text{Var}}(s_{lk}^{2})}{\sum_{k=1}^{K}{\left(s_{lk}^{2}-s_{l\text{median}}^{2}\right)}^{2}}\right).$$

Other moderated *t *statistics have been suggested and could be used instead, most notably the empirical Bayes *t *statistic used by Ji and Wong [7] and the moderated *t *of Smyth [19]. All of these approaches are designed to increase performance compared to the ordinary *t *statistic by incorporating information from all probes on the microarray into individual probe statistics. Here we choose the "shrinkage *t*" because it does not require any knowledge about the underlying distribution of probe values while providing similar performance compared to more complex models [18].

### 2.1 Hidden Markov Model

**Figure 1:**
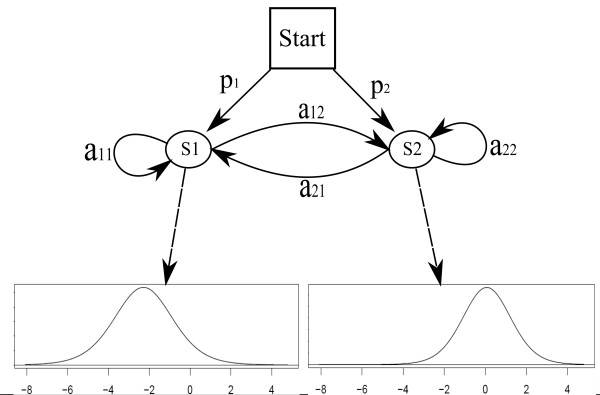
Hidden Markov model for the analysis of ChIP-chip tiling array data.

To detect enriched regions we use a two state discrete time hidden Markov model with continuous emission distributions and homogeneous transition probabilities (Figure 1), i.e., the transition probabilities depend only on the current state of the model. The use of homogeneous transition probabilities assumes equally-spaced probes within each observation sequence as well as a geometric distribution of the length of enriched regions. As discussed above there will be some variation in probe distances. Using a relatively small value for max_gap ensures that the assumption of homogeneity holds at least approximately. The two states of the model correspond to enrichment or no enrichment in the ChIP sample. The model is characterised by the set of states **S **= {*S*_1_, *S*_2_}, the initial state distribution *p*, the matrix of transition probabilities *A *and the state specific emission density functions *f*_*i*_, *i *= 1, 2. The emission distribution of state *S*_*i *_is modelled as a *t *distribution with location parameter *μ*_*i*_, scale parameter *σ*_*i*_, and *ν*_*i *_degrees of freedom.

The use of *t *distributions has the advantage that their sensitivity to outliers can be adjusted via the degrees of freedom parameter, making them more robust and versatile than normal distributions. This is particularly useful when *ν *is estimated from the data [20]. It should be noted that the *y*_*k *_modelled here are from a *t*-like distribution (Equation (1)). While this in itself might suggest the use of *t *distributions for the *f*_*i*_s, they are primarily chosen for their robustness. In the following we will refer to this model by its parameter vector *θ *= (*θ*_1_, *θ*_2_), where *θ*_1 _is the ordered pair (*p*, *A*) and *θ*_2 _the ordered triple (*μ*, *σ*, *ν*).

Given a hidden Markov model *θ *and an observation sequence *Y*, it is possible to compute the sequence of states *Q *= *q*_1_*q*_2_...*q*_*K *_that is most likely to produce *Y*. There are several approaches to obtaining *Q *[21]. Usually *Q *is computed either by maximising the posterior probabilities *P*(*q*_*k *_= *S*_*i*_|*Y*; *θ*), *k *= 1, ..., *K *or by calculating the sequence that maximises *P*(*Q|Y*; *θ*). The latter provides the single most likely sequence of states and can be computed efficiently by the Viterbi algorithm [22]. For the particular model used here both approaches are equivalent.

### 2.2 Parameter Estimation

In this section we will discuss two different approaches to estimate *θ *for the model described in Section 2.1. The methods under consideration are the EM algorithm, which is usually known as the Baum-Welch algorithm in the context of HMMs, and Viterbi training. While the Baum-Welch algorithm is guaranteed to converge to a local maximum of the likelihood function, it is computationally intensive. Viterbi training provides a faster alternative but may not converge to a local maximum.

#### 2.2.1 Initial Estimates

Both optimisation algorithms discussed here require initial parameter estimates. These are obtained from the data by first partitioning the vector of observations *Y *into two clusters using *k*-means clustering [23]. From these clusters the location and scale parameters of the corresponding states are obtained as the mean and variance of the observations in the cluster. In the following, *ν*_1 _= *ν*_2 _= 6 is used as initial estimate for the degrees of freedom parameters.

#### 2.2.2 Baum-Welch Algorithm

The Baum-Welch algorithm [16] is a well established iterative method for estimating parameters of HMMs. It represents the EM algorithm [24] for the specific case of HMMs. This algorithm can be used to optimise the transition parameters *θ*_1 _as well as the emission parameters *θ*_2_. Each iteration of the algorithm consists of two phases. During the first phase, the current parameter estimates are used to determine for each probe statistic in the observation sequence how likely it is to be produced by the different states of the model. In the second phase, parameters for the emission distributions of each state are estimated using contributions from all observations, according to the probability that they were produced by the respective state of the model. The state transition parameters are updated in a similar fashion, accounting for the probability of transitions between states based on the observation sequence and the current model. After each iteration this procedure results in a model which explains the observed data better than the previous one, approaching a locally optimal solution. Using this method parameter estimates are updated until convergence is achieved. The details of the resulting algorithm are outlined in Section 4.1.

This method of parameter estimation is computationally expensive and time-consuming for a typical tiling array data set. The computing time can be reduced by fixing the degrees of freedom for the emission distributions in advance, thus avoiding the root-finding required for the estimation of these parameters. While this does not provide the same flexibility as estimating the required degree of robustness from the data it reduces the complexity of the optimisation problem. It is noted by Liu and Rubin [25] that attempts to estimate the degrees of freedom are more likely to produce results which are of little practical interest. The impact on classification performance of this choice is investigated in Section 2.3.

The formulation of the Baum-Welch algorithm used in this article is based on the description given by Rabiner [21] and on the EM algorithm derived by Peel and McLachlan [26] for fitting mixtures of *t *distributions.

#### 2.2.3 Viterbi Training

While the Baum-Welch algorithm described in Section 2.2.2 is expected to provide good parameter estimates, it is computationally expensive. A faster model-fitting procedure can be devised by replacing the first phase of the Baum-Welch algorithm with a maximisation step. This method was introduced in [17] as segmental *k*-means and is now commonly referred to as Viterbi training. Unlike the Baum-Welch algorithm which allows each probe statistic to contribute to the parameter estimates for all states, Viterbi training assigns each observation to the state that is most likely to produce the given probe statistic. Thus each observation contributes to exactly one state of the model. While each iteration of this method is faster than one iteration of the Baum-Welch algorithm some iterations may decrease the likelihood of the model, thus failing to advance it towards a useful solution. See Section 4.2 for further details on the implementation of Viterbi training used here.

### 2.3 Testing

#### 2.3.1 Simulated Data

To assess the ability to distinguish between enriched and non-enriched probes of the models obtained by the different parameter estimation methods discussed in Section 2.2, we simulate data with known enriched regions. To ensure that the simulation study is providing meaningful results, it is based as closely on real data as possible. To this end, two independent analyses of the H3K27me3 data published by Zhang *et al*. [3] are carried out, one using TileMap [7], the other based on our model. The result of each analysis is used to generate a new dataset with known enriched regions. See Section 4 for further details. In the following these data are referred to as datasets I and II respectively. Since the simulation procedure is likely to bias results towards the model that was used in the process, we concentrate on the analysis of dataset I, with some results for dataset II presented for comparison. The use of data based on both models allows us to consider their performance under advantageous and disadvantageous conditions.

#### 2.3.2 Performance Measure

The performance of different models on these data is determined in terms of false positive and false negative rates at probe level. While the relative importance of false positives and false negatives depends on the experiment under consideration, they are often equally problematic in the context of ChIP-chip experiments, especially when considering experiments which investigate differences between different cell lines or developmental stages, where all incorrect classifications are of equal concern. In this context, we define false positives as probes that are classified as non-enriched by the analysis of the real data but are called enriched in the subsequent analysis of simulated data, and vice versa for false negatives.

The output of each model is the estimated posterior probability of enrichment for each probe. In practice, probe calls ("enriched" or "non-enriched") are generated from this posterior probability based on a 0.5 cut-off. For any given model, classification performance will change with the chosen threshold. Thus we assess model performance across a range of cut-offs, reporting the relative number of false positives and false negatives as well as the error rate. The latter is also used to determine the cut-off that minimises incorrect classification results, and model performance is judged on the numbers of incorrect classifications at this optimal cut-off and at the usual 0.5 cut-off, and on the distance between the optimal cut-off and 0.5. The trade-off between sensitivity and specificity provided by the different models is characterised with ROC curves and the associated AUC values.

Another measure of interest is the ability to characterise the length distribution of enriched regions correctly. When studying chromatin structure the extent of structural changes is of interest; this is the case for the data studied in Section 2.3.7. This property of the different models is investigated in Section 2.3.6.

#### 2.3.3 Estimating Degrees of Freedom

**Figure 2:**
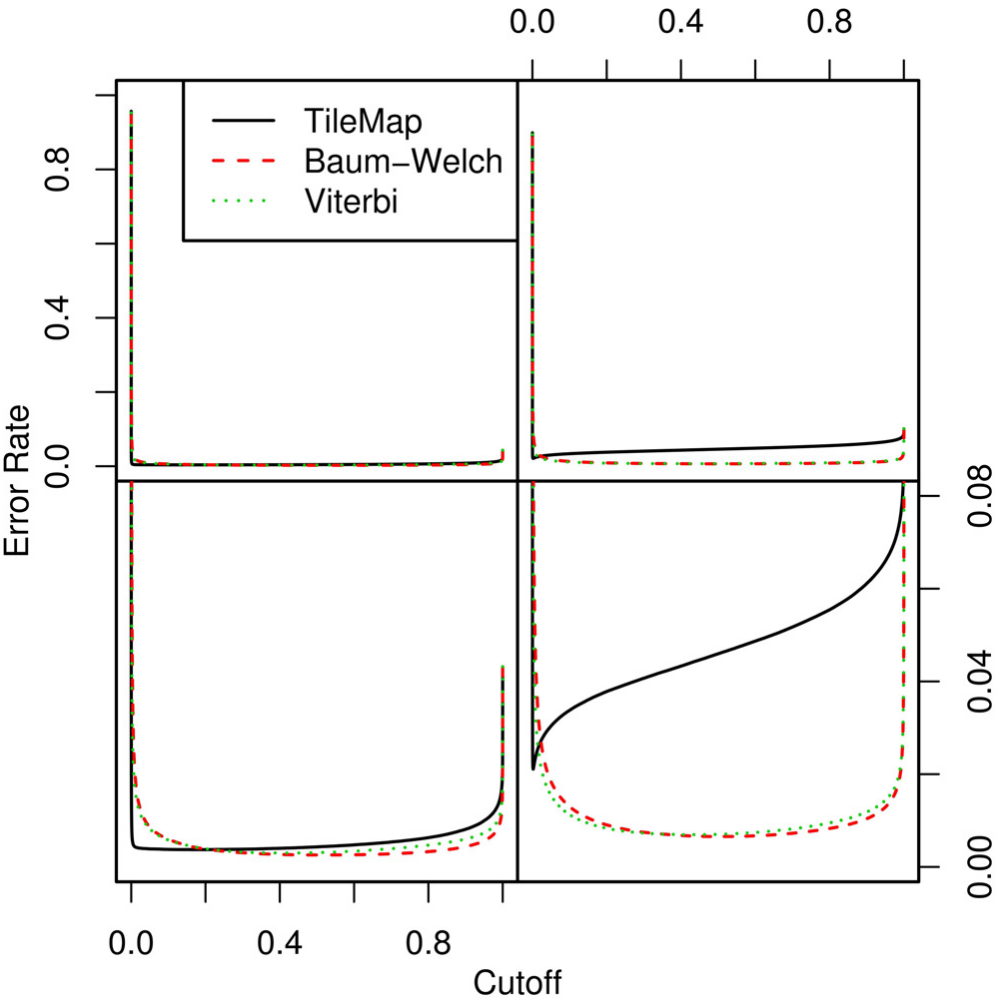
**Error rate for different models on datasets I and II**. Error rate resulting from the different models on dataset I (left) and II (right). When the total number of incorrect probe calls is considered, both parameter estimation procedures outperform TileMap on dataset I for cut-offs larger than 0.2. Both Baum-Welch and Viterbi training provide models with an optimal cut-off close to 0.5, while TileMap significantly underestimates the posterior probability resulting in an optimal cut-off of 0.19. The models with optimised parameters show similar performance on both datasets. On dataset II TileMap's performance is reduced in comparison to the results on dataset I. The main differences between the models considered here occur at error rates of 0–0.08. The relevant area of the figures in the top row is magnified in the plots below.

**Figure 3:**
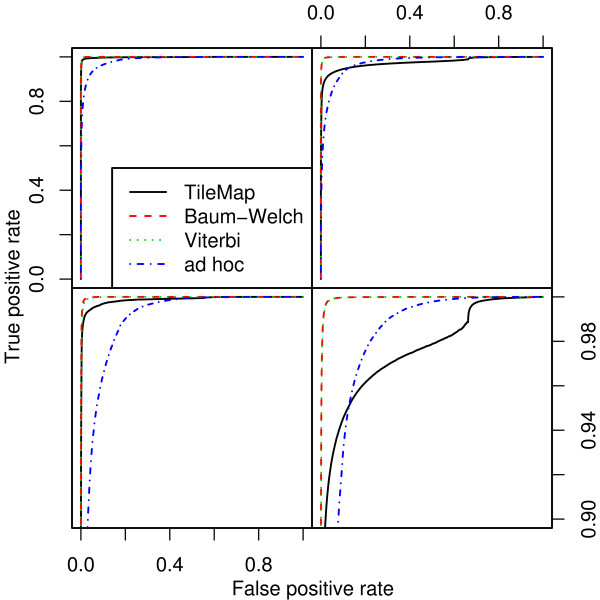
**ROC curves for different models on datasets I and II**. TileMap and the models with Baum-Welch and Viterbi training parameter estimates show similar performance on dataset I (left) with a small advantage for the models with optimised parameters. Comparison with a model using *ad hoc *parameter estimates highlights the performance increase achieved by optimising model parameters. On dataset II (right) TileMap performs similarly to the model with *ad hoc *parameter estimates. Figures on the bottom provide a close-up view of the plots above.

We now consider the performance of both the Baum-Welch procedure and Viterbi training when all model parameters, including the degrees of freedom *ν*, are estimated from the data. Both parameter estimation methods are used to fit an HMM to datasets I and II, and the performance of resulting models is assessed in terms of the achieved error rate (Figure 2), ROC curves (Figure 3) and their associated AUC (Table 1) for both datasets. To assess how well these methods perform in comparison to an established algorithm, we also fit a TileMap model to the two simulated datasets. The three models are compared to each other, as well as an *ad hoc *model which simply uses, without optimisation, the initial parameter estimates used by the two parameter optimisation methods. When comparing the performance of these models on both simulated datasets, it is important to consider that the simulation procedure introduces a bias towards the underlying model.

**Table 1:** AUC for different models on both simulated datasets.

	TileMap	Baum-Welch	Viterbi-Training	Viterbi-EM	ad hoc
dataset I	0.9986	0.9998	0.9997	0.9998	0.9869
dataset II	0.9749	0.9995	0.9994	0.9995	0.9728

All models with optimised parameters outperform TileMap on both simulated datasets. While TileMap performs well on dataset I it is only slightly better than the model with *ad hoc *parameter estimates.

Estimating all parameters from the data with either the Baum-Welch algorithm or Viterbi training leads to models with high sensitivity, producing fewer false negatives than TileMap for any given cut-off [see Additional file 1]. At the same time they lead to an increased number of false positives [see Additional file 2] compared to TileMap, indicating a slight reduction of specificity. When considering the error rate it becomes apparent that both Baum-Welch and Viterbi training provide a favourable trade-off between sensitivity and specificity. These models reduce the number of incorrect classifications compared to TileMap both at the usual 0.5 cut-off and at the optimal cut-off. Moreover, while the Baum-Welch algorithm and Viterbi training both lead to models with an optimal cut-off close to 0.5 (0.51 and 0.42 respectively), TileMap provides an optimal cut-off of 0.19, indicating that it underestimates the posterior probability of enrichment. This becomes even more apparent when considering the result for dataset II where the optimal cut-off for TileMap is at 0.002 compared to 0.5 for Baum-Welch and 0.41 for Viterbi training. This result suggests that TileMap is more tuned towards avoiding false positives than false negatives. From the above results we estimate that the weight given to false positives by TileMap is approximately 3.2 and 26 times larger than the weight for false negatives on datasets I and II respectively. The ROC curves (Figure 3) provide further evidence that the models with MLEs outperform TileMap. Although all three models perform well on dataset I, both parameter optimisation methods lead to better results than TileMap. The benefits of optimising parameter estimates are further highlighted by the performance of the model with *ad hoc *estimates that is used as starting point for the optimisation procedures. On both datasets, optimised parameters provide a notable increase in performance, with TileMap performing only slightly better than the *ad hoc *model on dataset II.

#### 2.3.4 Fixed Degrees of Freedom

**Figure 4:**
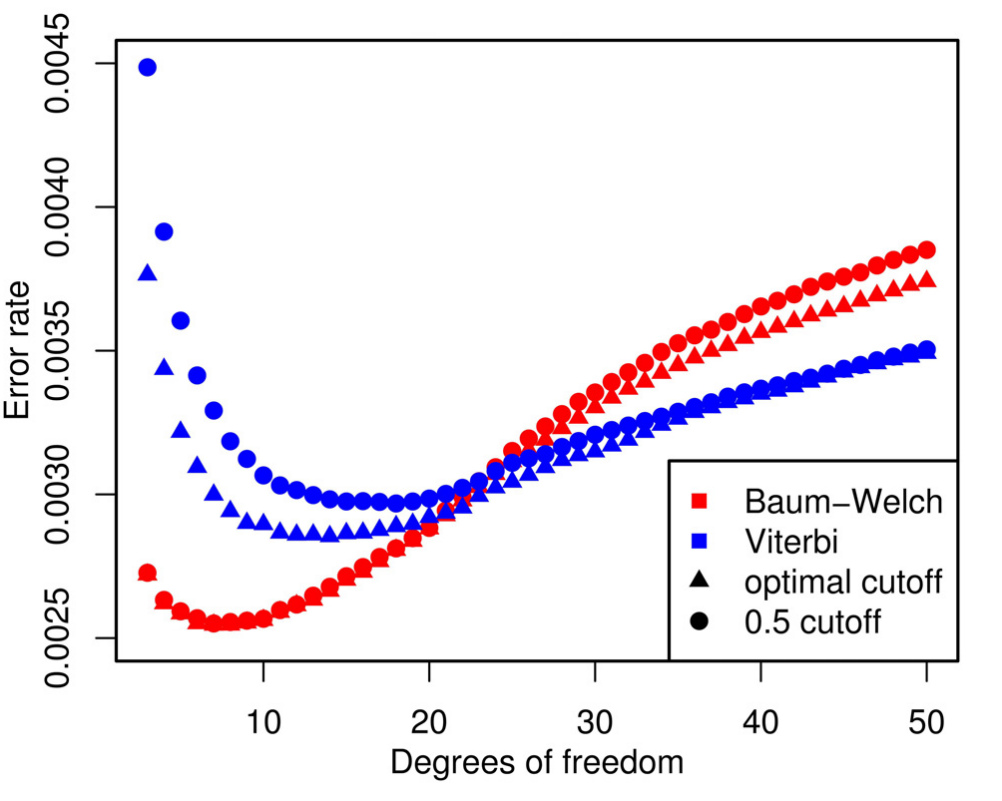
**Model performance for different choices of *ν***. The Baum-Welch model (red) performs better for relatively small values of *ν *while Viterbi training (blue) favours larger *ν*. For the optimal choice of *ν *the Baum-Welch parameter estimates lead to an optimal cut-off close to 0.5.

**Figure 5:**
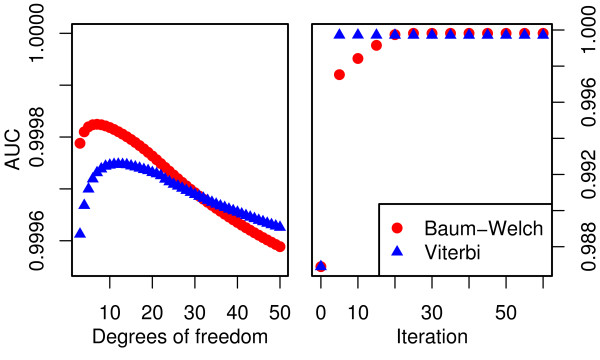
**AUC for different choices of *ν *and increasing numberof iterations**. Change in AUC for different choices of *ν *(left). The Baum-Welch model performs better for relatively small values of *ν *while Viterbi training favours larger *ν*. Improvements in AUC with increasing number of iterations (right). The performance of the Viterbi trained model improves substantially during the first five iterations. Further iterations only produce small changes in the AUC. The Baum-Welch method requires more iterations to obtain the same AUC as as the Viterbi model. After 20 iterations the Baum-Welch model starts to outperform the Viterbi model.

Estimating *ν*, the degrees of freedom, for *t *distributions from the data is time-consuming and may not be very accurate, especially for relatively large values of *ν*. In this section we investigate the effect of fixing *ν *a priori for both states of the model. Only the case *ν*_1 _= *ν*_2 _is considered here. The remaining parameters are estimated from the training data using the Baum-Welch algorithm and Viterbi training with *ν *= 3, 4, ..., 50. For each value of *ν*, we report the error rate (Figure 4) as well as the AUC (Figure 5) on the simulated data.

For the best combination of *ν *and cut-off, both parameter estimation methods result in models with a classification performance comparable to the case of variable degrees of freedom (Figure 2). While the Baum-Welch algorithm tends to produce models with an optimal cut-off close to 0.5, Viterbi training only achieves this for large values of *ν*. Notably, the best classification performance of the Viterbi trained model is achieved with 14 degrees of freedom and a 0.37 cut-off compared to 7 degrees of freedom and a 0.49 cut-off from Baum-Welch. This results in a decreased performance of the Viterbi model relative to the Baum-Welch model at the 0.5 cut-off.

#### 2.3.5 Convergence

**Figure 6:**
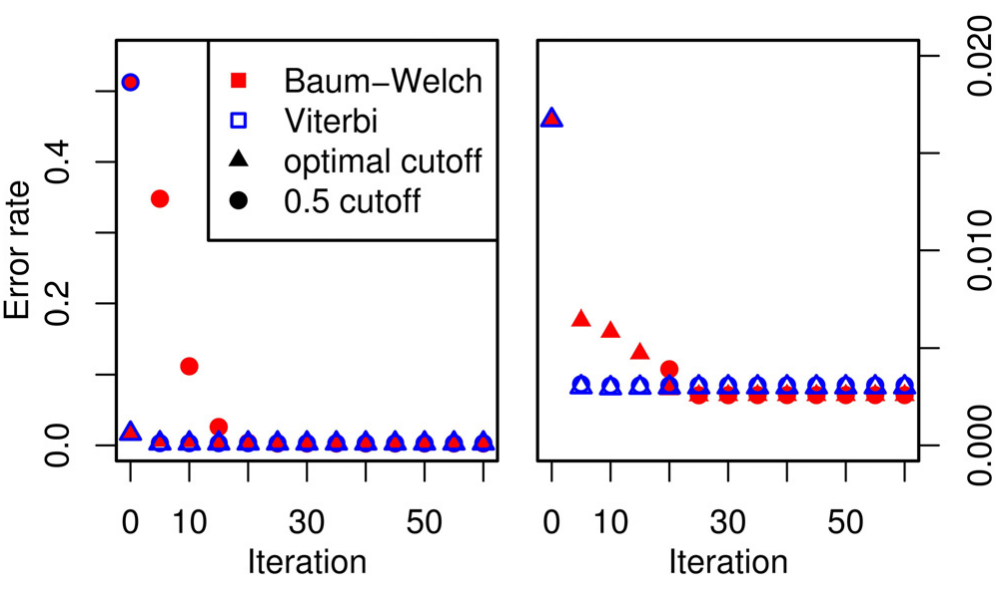
**Error rate at optimal and 0.5 cutoff for increasing number of iterations**. Parameter estimates obtained by the Baum-Welch algorithm (filled symbols) and Viterbi training (open symbols) improve model performance with increasing nuber of iterations. Viterbi training quickly approaches its optimal solution and initially outperforms Baum-Welch. The final model produced by the Baum-Welch algorithm provides a lower error rate than Viterbi training.

To reduce the time required for parameter estimation it is useful to limit the number of iterations. While each iteration of the Baum-Welch algorithm is guaranteed to improve the likelihood of the model, small changes to the parameter values do not necessarily lead to significant changes in the classification result. Furthermore, Viterbi training is not guaranteed to converge to a local maximum of the likelihood function and a likelihood based convergence criterion may not be appropriate for this method. Here we investigate the convergence of both algorithms based on the error rate and AUC to gauge the number of iterations required to achieve good classification results. Parameter estimation is performed with 60 iterations for both algorithms. Current estimates are used to classify the test data at every 5^th ^iteration and AUC (Figure 5) and error rate (Figure 6) are determined.

The most striking difference in the convergence behaviour of the two methods is that Viterbi training appears to obtain good parameter estimates within a small number of iterations. Further iterations of the algorithm do not improve results substantially, whereas the Baum-Welch procedure provides parameter estimates that are better than the ones obtained by Viterbi training, both in terms of likelihood and classification performance, but takes substantially longer to obtain these estimates. The Baum-Welch algorithm not only requires more iterations than Viterbi training, but the time required for each iteration is also longer.

#### 2.3.6 Length Distribution of Enriched Regions

**Figure 7:**
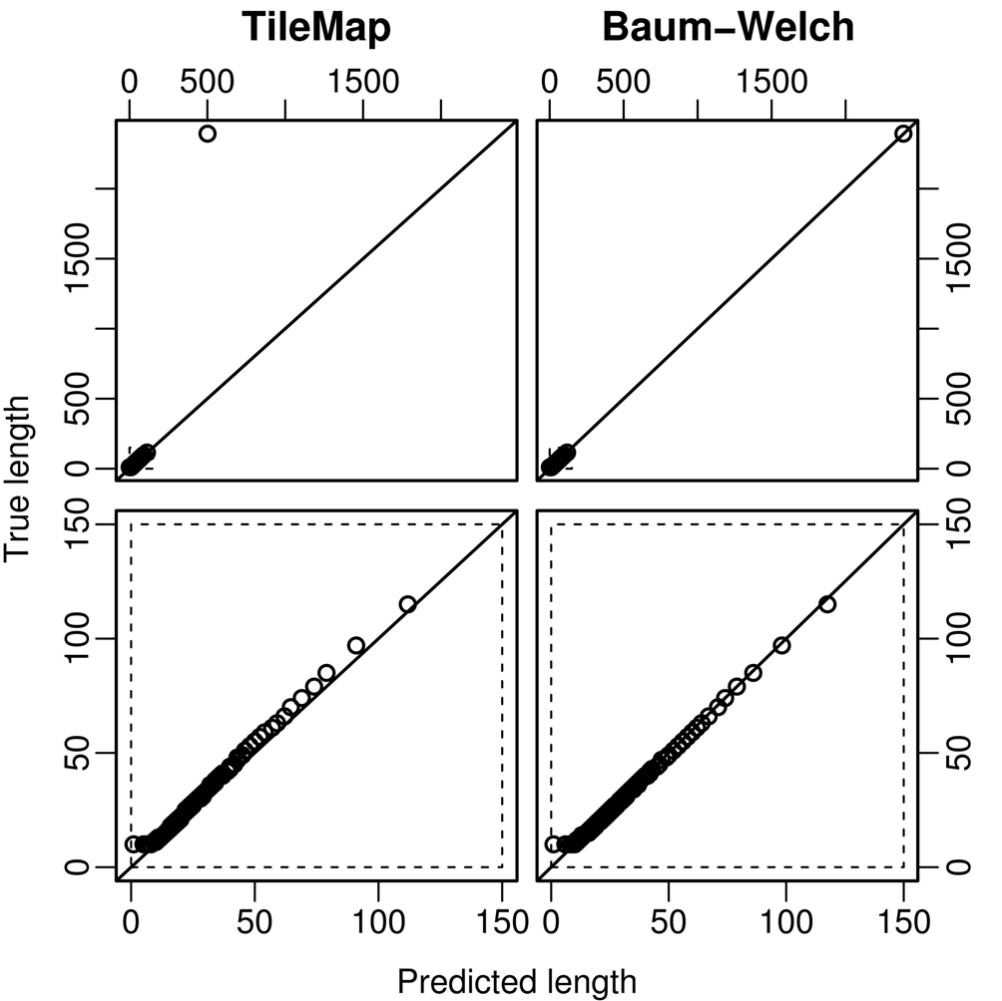
**Length distribution of enriched regions from dataset I**. Quantile-quantile plots comparing length distributions of enriched regions found with TileMap (left) and with the model based on maximum likelihood estimates (right) to the true length distribution of enriched regions in dataset I. Figures on the bottom provide a close-up view of the plots above. Each dot represents a percentile of the length distributions.

**Figure 8:**
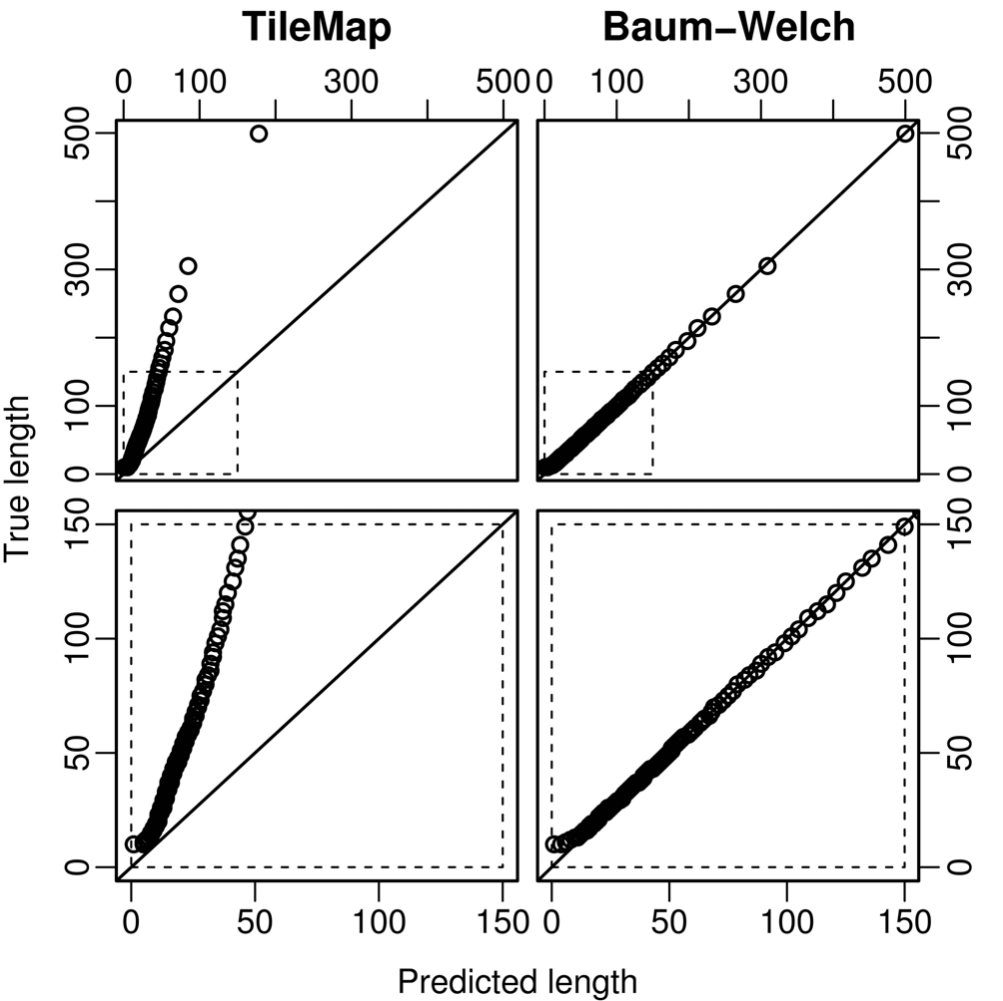
**Length distribution of enriched regions from dataset II**. Quantile-quantile plots comparing length distributions of enriched regions found with TileMap (left) and with the model based on maximum likelihood estimates (right) to the true length distribution of enriched regions in dataset II. Figures on the bottom provide a close-up view of the plots above. Each dot represents a percentile of the length distributions.

When studying histone modifications one possible characteristic of interest is the length of enriched regions. To assess how accurately the different methods reflect the length distribution of enriched regions, we compare the length of regions predicted by TileMap and by the model (using Baum-Welch parameter estimates) to the length distribution of enriched regions in the simulated data (the "true length distribution"). Note that this length distribution may vary from the one found in real data. Nevertheless this comparison highlights some of the differences between the two models. Quantile-quantile plots of the respective length distributions show that TileMap systematically underestimates the length of enriched regions (Figure 7 (bottom left) and Figure 8 (bottom left)). While this effect is relatively small on dataset I there is some indication that it increases with region length and long regions may not be characterised appropriately by TileMap (Figure 7 (top left)). This observation is further supported by the length distribution of enriched regions produced by TileMap on dataset II (Figure 8 (left)). Enriched regions in dataset II are generally longer than regions in dataset I. This difference is not captured by TileMap. Both TileMap and the Baum-Welch trained model produce several regions that are shorter than the shortest enriched region in the simulated data (Figure 7 (bottom)). There are two possible explanations for these short regions. They may be caused by underestimating the length of enriched regions, possibly splitting one enriched region into several predicted regions, or they may represent spurious enriched results produced by the model. In each case there is the possibility that the occurrence of extremely short regions is caused either by an intrinsic shortcoming of the model or by artifacts introduced during the simulation process. Since the simulation relies on TileMap to identify enriched and non-enriched probes it is inevitable that some probes will be misclassified. Subsequently these probes may be included in the simulated data, causing short disruptions of enriched and non-enriched regions. A sufficiently sensitive model could detect these unintended changes between enriched and non-enriched states.

To investigate further which of these is the case, we first examine the number of enriched probes contained in the short regions found by the Baum-Welch model and by TileMap respectively. The model with Baum-Welch parameter estimates found 126 regions with less than 10 probes. These regions contain a total of 866 probes of which 717 are in enriched regions. While this indicates that the majority of short regions is due to underestimating the length of enriched regions, several spurious probe calls remain. TileMap produced 249 regions with less than 10 probes, containing a total of 1781 probes, of which 1753 are in enriched regions. This is strong evidence that almost all of these short regions are caused by underestimating the length of enriched regions, and is consistent with the above observation that TileMap systematically underestimates the length of enriched regions.

To investigate whether the spurious short regions produced by the Baum-Welch model are due to an intrinsic shortcoming of the model or are artifacts introduced by the simulation procedure, we turn to real data. Here we focus on enriched regions containing only a single probe, which are most likely to be false positives. On dataset I the Baum-Welch model produced six of these extremely short regions. One of these probes is a true positive from an enriched region containing ten probes, i.e., the length of this region is underestimated by the Baum-Welch model. Of the remaining five probes three are identical, leaving three unique probes to be investigated further. For each of these three probes, we determine its position in the real data and its distance from enriched regions identified by TileMap and by our model (Section 2.3.7). Two of the probes are found to be located close to enriched regions identified by TileMap (142 and 391 bp) and all three probes are contained within enriched regions identified by our model [see Additional file 3]. This suggests that these probes may have been misclassified by TileMap during the original analysis, leading to an overestimation of the number of false positives produced by the Baum-Welch model on dataset I.

#### 2.3.7 Application to ChIP-Chip Data

To investigate the performance of our model further, we apply it to the data of [3] and compare the result to the original analysis. Based on the results of the simulation study (Sections 2.3.3–2.3.6) we use the following procedure:

1. Quantile normalise and log transform data;

2. Calculate probe statistics (Equation (2));

3. Obtain initial estimates (Section 2.2.1);

4. Use 5 iterations of Viterbi training to improve initial estimates;

5. Use 15 iterations of Baum-Welch algorithm to obtain maximum likelihood estimates;

6. Apply resulting model to data to identify enriched regions.

**Figure 9:**
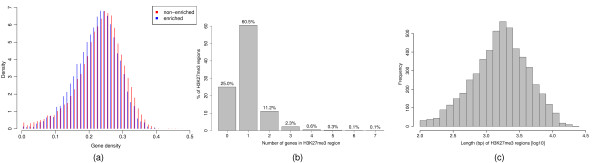
**Analysis of ChIP-chip data**. (a) Gene density in areas surrounding genes that contain H3K27me3 enriched regions and genes that do not contain enriched regions. (b) Number of genes found in H3K27me3 regions. While most enriched regions cover a single gene, there is a substantial number of H3K27me3 regions that cover several genes and enriched regions are found to contain up to seven genes. (c) Length distribution of H3K27me3 regions.

This results in the detection of 5285 H3K27me3 regions covering 12.9 Mb of genomic sequence. Of these enriched regions, 3962 (~75%) are overlapping at least one annotated transcript. A total of 4982 or about 18.9% of all annotated genes are found to be enriched for H3K27me3. While most of the enriched regions cover a single gene, some regions are found to contain up to seven genes (Figure 9(b)). Enriched regions are predominantly longer than 1 kb with some extending over more than 20 kb (Figure 9(c)).

To assess whether there is a difference between regions of the genome that show H3K27me3 enrichment and the rest of the genome, we investigate the density of genes in the neighbourhood of genes that appear to be regulated by H3K27me3, and compare this to the gene density in other regions of the genome. For this purpose we obtain the gene density for the 50 kb upstream and downstream of each gene as (bp annotated as genes)/100 kb. The resulting gene densities for genes with and without enriched regions are summarised in Figure 9(a). There are visible differences between the two distributions which we test for significance with a two sided Kolmogorov-Smirnov test; this results in an approximate *p*-value of 2 × 10^-15^. The significance of this result is further confirmed by a resampling experiment: the smallest *p*-value obtained from a series of 10000 resampled datasets is 1 × 10^-6^.

## 3 Conclusion

With the use of MLEs for all model parameters, our model clearly improves classification performance on simulated data compared to *ad hoc *estimates, and outperforms TileMap. While our model produced some short regions that appear to be false positives, they are readily explained as a result of the simulation process. Comparison of results on simulated and real data suggests that TileMap produced a large number of false negatives in the original analysis used as the basis for the simulation. Inevitably, these false negatives were selected as part of non-enriched regions during the simulation process. The fact that the model with Baum-Welch parameter estimates was able to identify these isolated enriched probes despite the non-enriched contexts where they appeared emphasises the high sensitivity of the model.

TileMap's apparent tendency to penalise false positives more than false negatives clearly contributes to its relatively low performance in our comparisons which are based on the assumption that both types of error are equally problematic. While this is the case for the application considered here, one may argue that false positives are indeed of greater concern in some cases. When this is the case, TileMap's trade-off between sensitivity and specificity may lead to better results. However, it should be noted that the relative weights given to false positives and false negatives by TileMap can vary substantially between datasets. The parameter estimation procedure used for our model on the other hand provides consistent performance at the chosen cut-off.

The model-fitting procedure derived from the results of the simulation study (Sections 2.3.3–2.3.6) provides a fast and reliable approach to parameter estimation. This method retains all the favourable properties of the Baum-Welch algorithm while utilising the reduced computing time provided by Viterbi training. The use of MLEs ensures that model parameters are appropriate for the data. Results from the simulation study show that estimating model parameters from the data improves the model's ability to recognise enriched regions of varying length and generally improves classification performance.

### 3.1 Future Work

**Figure 10:**
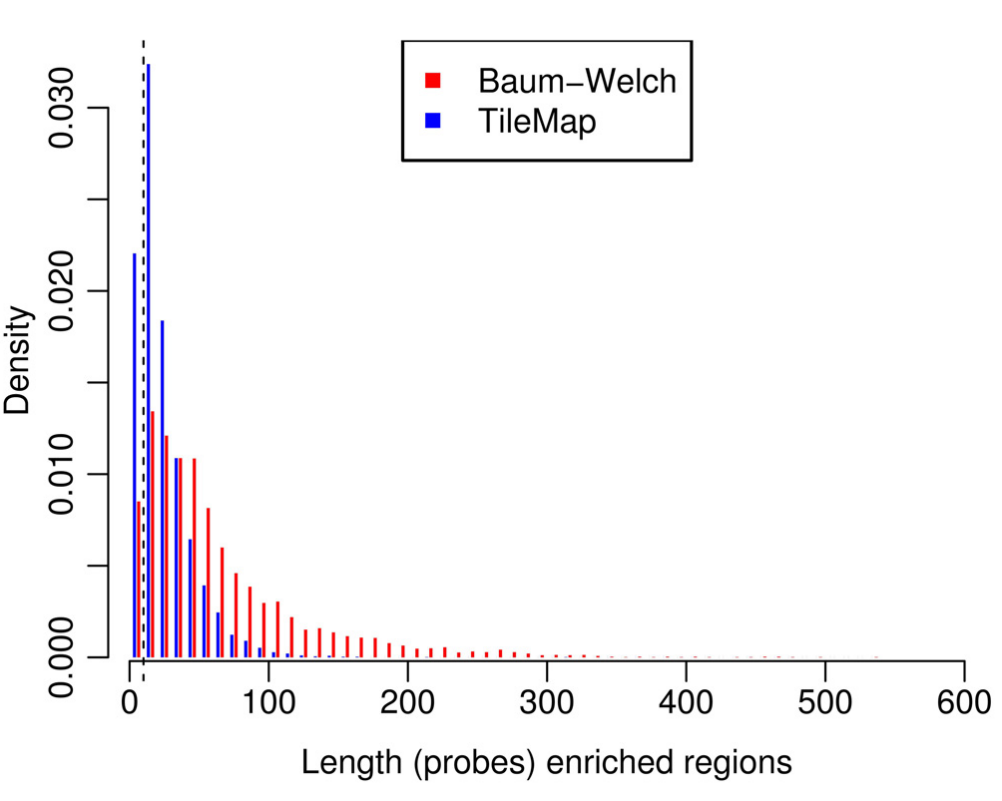
**Length distribution of enriched regions from real data**. Length distribution of enriched regions as determined by TileMap (blue) and Baum-Welch (red). Region length is determined in terms of probes per region. Both distributions were truncated at 10 for the simulation, ensuring that all regions in the simulated data contain at least ten probes.

The analysis of the H3K27me3 data (Section 2.3.7) largely confirms the analysis of [3] although there are some notable differences. Most importantly, the H3K27me3 regions detected by our analysis are longer than the ones determined by TileMap (Figure 10). While Zhang *et al*. [3] found few regions longer than 1 kb, our analysis indicates that over 70% of enriched regions have a length of at least 1 kb, with the longest region spanning over 20 kb. Accordingly we find more regions that extend over several genes (Figure 9(b)). This may have implications for conclusions about the spreading of H3K27me3 regions in *Arabidopsis*.

At this stage, the biological significance of the observed difference in gene density in the neighbourhood of enriched and non-enriched genes is unclear. However, it indicates that the two groups of genes differ in a significant way. This suggests that the partition into enriched and non-enriched genes produced by our analysis is indeed meaningful.

The hidden Markov model presented in this article uses homogeneous transition probabilities, assuming that all probes are spaced out equally along the genome. To satisfy this assumption at least approximately, we use a fixed cut-off of 200 bp to partition the sequence of probe statistics such that there are no large gaps between probes. This arbitrary cut-off could be avoided by using a continuous time hidden Markov model.

## 4 Methods

### 4.1 Baum-Welch Algorithm

The Baum-Welch algorithm [16] used to estimate parameters for our model is outlined in Section 2.2.2; further details are given below. Computing the likelihood of the long observation sequences produced by tiling arrays involves products of many small contributions. This typically results in likelihoods below machine precision. To avoid this effect computations are carried out in log-space, using the identity

(4) ln(*x *+ *y*) = ln(*x*) + ln (1 + *e*^ln(*y*)-ln(*x*)^).

In the following we use ^ln^∑ to denote summations which should be computed via Equation (4). The sequence of probe statistics *Y *is split into *D *observation sequences *Y *^(*d*) ^such that the distance between probes within each observation sequence is at most max_gap and the distance between the end points of different observation sequences is greater than max_gap.

The emission distribution of state *S*_*i *_is given as

(5) $$f_{i}(y_{k};\mu_{i},\sigma_{i},\nu_{i})=\frac{\Gamma \left(\frac{\nu_{i}+1}{2}\right)\Gamma {\left(\frac{\nu_{i}}{2}\right)}^{-1}}{\sigma_{i}\sqrt{\pi \nu_{i}{\left(1+\frac{{\left(y_{k}-\mu_{i}\right)}^{2}}{\sigma_{i}\nu_{i}}\right)}^{\nu_{i}+1}}}.$$

For a given parameter set *θ *we can obtain new parameter estimates for transition probabilities by calculating

(6) $$\xi_{kij}^{d}=\ln[P(q_{k}^{d}=S_{i},q_{k+1}^{d}=S_{j}|Y^{\left(d\right)};\theta )]$$

(7) $$\begin{array}{ll} = & \alpha_{ki}^{d}+\ln[a_{ij}]+\ln[f_{i}(y_{k+1}^{d};\theta_{2})]+\\ & +\beta_{(k+1)j}^{d}-\ln[P(Y^{\left(d\right)};\theta )]. \end{array}$$

Here *α*_*k *_and *β*_*k *_are known as forward and backward variables. For observation sequence *d*, *d *= 1, ..., *D*, they are defined as

(8) $$\alpha_{1i}^{d}=\ln[p_{i}]+\ln[f_{i}(y_{1}^{d};\theta_{2})],$$

(9) $$\begin{array}{lll} \alpha_{(k+1)j}^{d} & = & \left(\ln\sum_{i=1}^{N}\left(\alpha_{ki}^{d}+\ln[a_{ij}]\right)\right)\\ & & +\ln[f_{j}(y_{k+1}^{d};\theta_{2})], \end{array}$$

where 1 ≤ *i *≤ *N*, 1 ≤ *j *≤ *N*, 1 ≤ *k *<*K*_*d *_and

(10) $$\beta_{K_{d}i}^{d}=0,$$

(11) $$\beta_{ki}^{d}=\begin{array}{c} \ln \end{array}\sum_{j=1}^{N}\left(\ln[a_{ij}]+\ln[f_{j}(y_{k+1}^{d};\theta_{2})]+\beta_{(k+1)j}^{d}\right),$$

where 1 ≤ *i *≤ *N*, 1 ≤ *i *≤ *N*, *k *= *K*_*d *_- 1, ..., 1. Note that ln [*P*(*Y *^(*d*)^; *θ*)] is given by $\begin{array}{c} \ln \end{array}\sum_{i=1}^{N}\alpha_{K_{d}}^{d}$ We then calculate

(12) $$\gamma_{ki}^{d}=\ln[P(q_{k}^{d}=S_{i}|Y^{\left(d\right)};\theta )]$$

(13) $$=\alpha_{ki}^{d}+\beta_{ki}^{d}-\ln[P(Y^{\left(d\right)};\theta )]$$

(14) $$=\begin{array}{c} \ln \end{array}\sum_{j=1}^{N}\xi_{kij}^{d}.$$

Combining the estimates from all observation sequences we obtain new parameter estimates for the transition probabilities:

(15) $$\ln[{\hat{a}}_{ij}]=\begin{array}{c} \ln \end{array}\sum_{d=1}^{D}\begin{array}{c} \ln \end{array}\sum_{k=1}^{K_{d}-1}\xi_{kij}^{d}-\begin{array}{c} \ln \end{array}\sum_{d=1}^{D}\begin{array}{c} \ln \end{array}\sum_{k=1}^{K_{d}-1}\gamma_{ki}^{d},$$

(16) $$\ln[{\hat{p}}_{i}]=\begin{array}{c} \ln \end{array}\sum_{d=1}^{D}\gamma_{1i}^{d}-\ln[D].$$

Calculations for the re-estimation of *θ*_2 _may involve negative values and cannot be carried out in log-space.

To obtain the required parameter estimates we first define $\ln[\tau_{ki}^{d}]=\gamma_{ki}^{d}$ and then compute

(17) $$u_{ki}^{d}=\frac{\nu_{i}+1}{\nu_{i}+{\left(y_{k}^{d}-\mu_{i}\right)}^{2}},$$

(18) $${\hat{\mu }}_{i}=\frac{\sum_{d=1}^{D}\sum_{k=1}^{K_{d}}\tau_{ki}^{d}u_{ki}^{d}y_{k}^{d}}{\sum_{d=1}^{D}\sum_{k=1}^{K_{d}}\tau_{ki}^{d}u_{ki}^{d}},$$

(19) $${\hat{\sigma }}_{i}=\frac{\sum_{d=1}^{D}\sum_{k=1}^{K_{d}}\tau_{ki}^{d}u_{ki}^{d}{\left(y_{k}^{d}-{\hat{\mu }}_{i}\right)}^{2}}{\sum_{d=1}^{D}\sum_{k=1}^{K_{d}}\tau_{ki}^{d}}.$$

There is no closed form estimate for *ν*_*i*_. To obtain ${\hat{\nu }}_{i}$ one has to find a solution to the equation

(20) $$\begin{array}{l} {}[-\psi \left(\frac{\nu_{i}}{2}\right)+\ln\left(\frac{\nu_{i}}{2}\right)+1+\\ +\frac{1}{\sum_{k=1}^{K_{d}}\tau_{ki}^{d}}\sum_{k=1}^{K_{d}}\tau_{ki}^{d}\left(\ln\left(u_{ki}^{d}\right)-u_{ki}^{d}\right)+\\ +\psi \left(\frac{\nu_{i}+1}{2}\right)-\ln\left(\frac{\nu_{i}+1}{2}\right)]=0 \end{array}$$

where *ψ *is the digamma function. Standard root-finding techniques are employed to find a solution to (20).

### 4.2 Viterbi Training

Viterbi training provides a faster alternative to the Baum-Welch algorithm. See Section 2.2.3 for a high level description of the algorithm. Details of the parameter estimation procedure are given below. Instead of calculating the conditional expectation of the complete data log likelihood, this algorithm first computes the most likely state sequence *Q *given the observation sequence *Y *and the current model *θ*. The sequence *Y *is partitioned according to *Q*, assigning each observation to the state that it most likely originated from. New estimates for *θ*_1 _are then obtained by calculating

(21) $${\hat{p}}_{i}=\frac{1}{D}|\{d=1,...,D:q_{1}^{d}=S_{i}\}|,$$

(22) $${\hat{a}}_{ij}=\frac{\left|\{d=1,...,D:q_{k}^{d}=S_{i}\text{ and }q_{k+1}^{d}=S_{j}\}\right|}{\sum_{d=1}^{D}\left(K_{d}-1\right)}.$$

Updates for *μ *and *σ *are obtained as in Section 2.2.1. The degrees of freedom *ν *can be either fixed in advance or estimated from the data using Equation (20) by setting $\tau_{ki}^{d}=1$ if $\left(q_{ki}^{d},q_{k+1}^{d})=(S_{i},S_{j}\right)$ and $\tau_{ki}^{d}=0$ otherwise.

### 4.3 Simulated Data

In a first step following the original analysis by [3], TileMap [7] is used with the HMM option to define enriched and non-enriched probes. Note that, although this classification of probes is not perfect, it can be assumed that most probes are assigned to the correct group. The length distribution of enriched and non-enriched regions detected by TileMap is used to determine the length distributions for the simulated data after removing all regions that contain less than 10 probes (Figure 10). Data are generated by first determining the length of enriched and non-enriched regions from the empirical length distributions and then sampling data points from the respective TileMap generated clusters. Following this procedure, 600 sequences with one to ten enriched regions in each sequence are generated. A second dataset is generated by applying the model described in Section 2. Note that, although this procedure relies on the classifications produced by the respective models, the resampling procedure will place individual probe values in a new context of surrounding probes, which may lead to different probe calls in the analysis of the simulated data. Prior to analysis all data are quantile normalised.

## 5 Availability

The parameter estimation methods used in this article are available as part of the R package tileHMM from the authors' webpage  and from CRAN. The simulated data used in this study is available from the authors' web page.

## 6 Authors' contributions

PH conducted the research and wrote the manuscript. DB critically revised the manuscript. GS conceived the project. DB and GS provided supervision to PH. All authors have read and approved the final manuscript.

## Supplementary Material

Additional file 1 **False negative probe calls resulting from different models**. For any given cut-off TileMap produces more false negatives than the Baum-Welch and Viterbi trained models. Click here for file

Additional file 2 **False positive probe calls resulting from different models**. For any given cut-off TileMap produces fewer false positives than the Baum-Welch and Viterbi trained models. Click here for file

Additional file 3 **Origin of isolated enriched probes in dataset I**. The isolated enriched probes identified in dataset I by the Baum-Welch model originate from enriched regions identified by the Baum-Welch model in the real data. Two out of three probes are located close to enriched regions identified by TileMap. Click here for file
