# Supplementary figures and images for: Parameter estimation for robust HMM analysis of ChIP-chip data
Source: BMC Bioinformatics. 2008 Aug 18;9:343. doi: 10.1186/1471-2105-9-343 (PMC2536674; doi:10.1186/1471-2105-9-343)

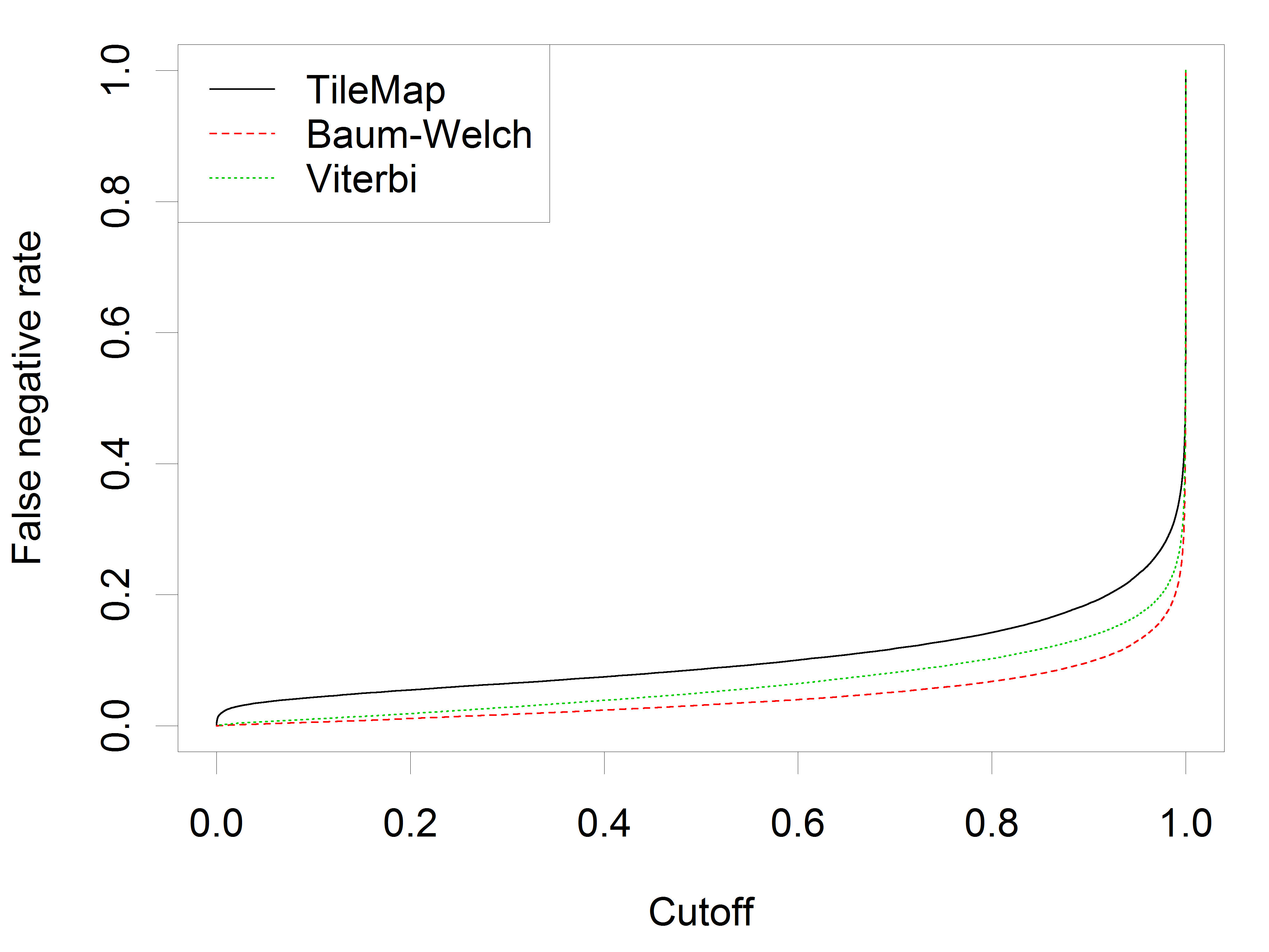

Supplement: Additional file 1 — False negative probe calls resulting from different models. For any given cut-off TileMap produces more false negatives than the Baum-Welch and Viterbi trained models. [file 1471-2105-9-343-S1.png]

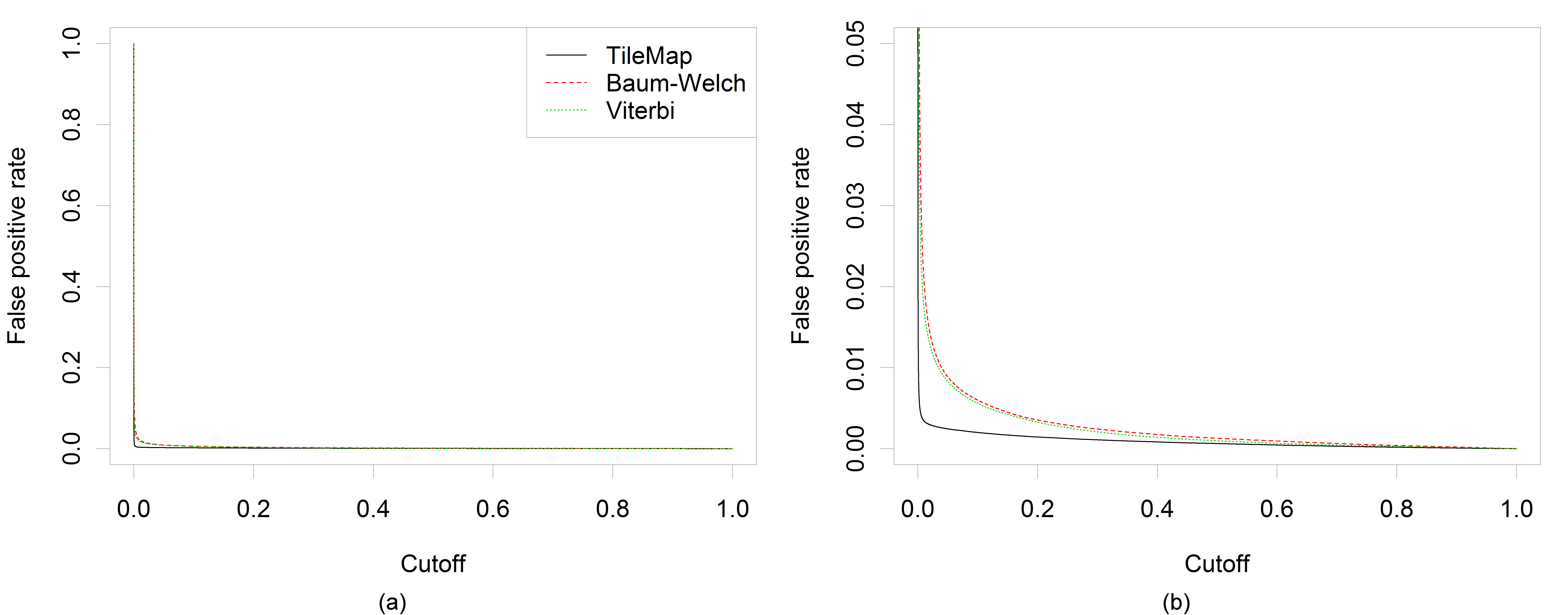

Supplement: Additional file 2 — False positive probe calls resulting from different models. For any given cut-off TileMap produces fewer false positives than the Baum-Welch and Viterbi trained models. [file 1471-2105-9-343-S2.png]

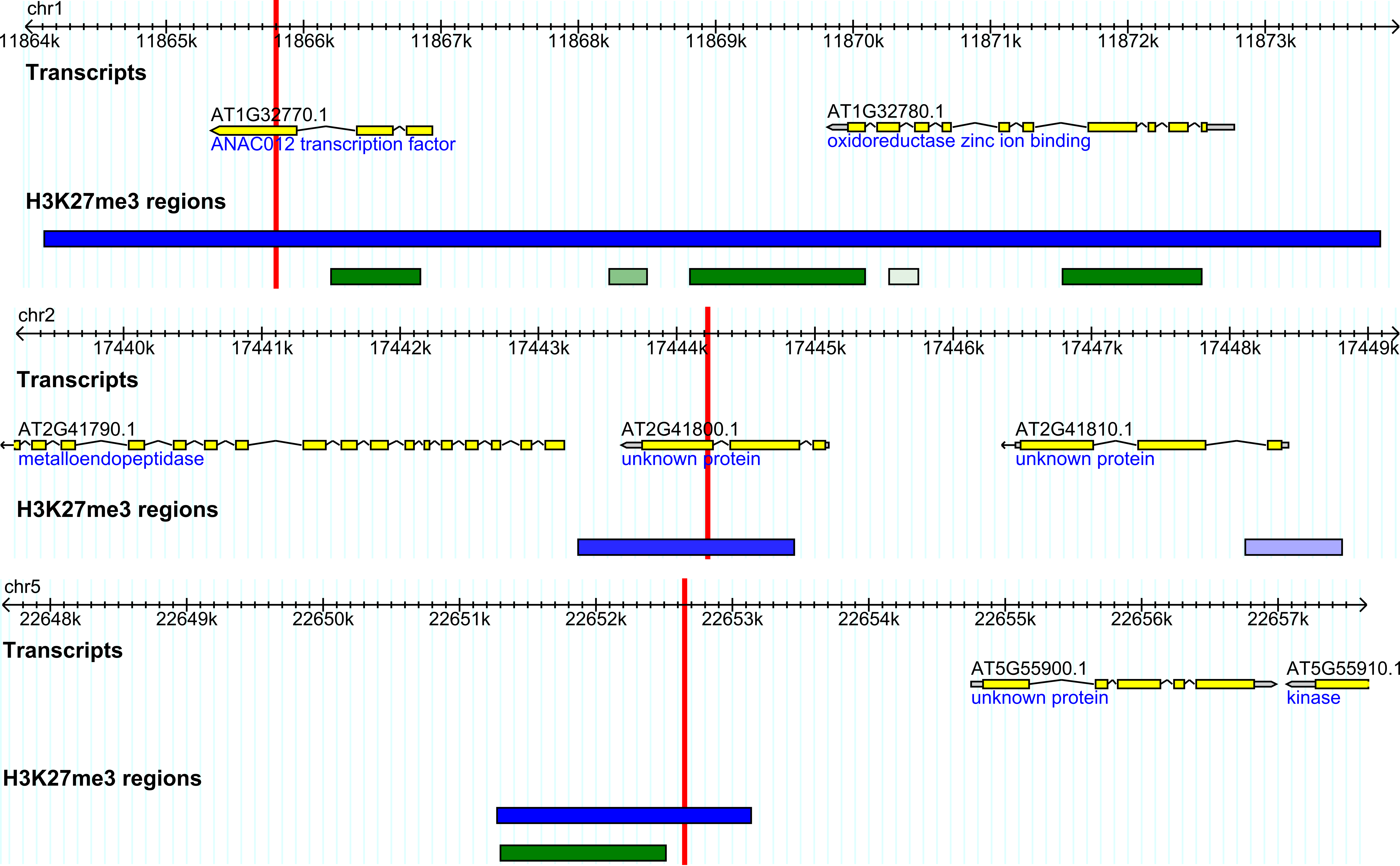

Supplement: Additional file 3 — Origin of isolated enriched probes in dataset I. The isolated enriched probes identified in dataset I by the Baum-Welch model originate from enriched regions identified by the Baum-Welch model in the real data. Two out of three probes are located close to enriched regions identified by TileMap. [file 1471-2105-9-343-S3.png]
